# Prediction of surgery type for uterine fibroids using machine learning algorithms and hormone values

**DOI:** 10.1097/MD.0000000000047965

**Published:** 2026-03-06

**Authors:** İnci Öz, Ali Utku Öz

**Affiliations:** a Department of Gynaecology of Obstetrics, Medicana Atakoy Hospital, Istanbul, Turkey; b Department of Gynaecology of Obstetrics, Başakşehir Çam and Sakura City Hospital, Istanbul, Turkey.

**Keywords:** artificial intelligence, decision modeling, female sex hormones, fibroid characteristics, hysterectomy, machine learning, myomectomy, uterine fibroid

## Abstract

This study aimed to develop and externally validate machine learning (ML)-based models to characterize surgical classification patterns between hysterectomy and myomectomy using fibroid characteristics and female sex hormone profiles. This multicenter study included 600 women with uterine fibroids (UFs) who presented to 3 hospitals. Of these, 362 (60.3%) underwent hysterectomy, while 238 (39.7%) underwent myomectomy. Statistical analyses and ML models were applied to both groups. ML model development was performed using individual and combined inputs of female sex hormones together with fibroid characteristics. Five ML classification algorithms were evaluated, including support vector machines, decision trees, random forests, k-nearest neighbors, and logistic regression. In total, 2555 model–input combinations were tested. The performance of the selected best-performing model was further evaluated using an independent, blinded external validation cohort comprising 30 cases. Women in the hysterectomy group had significantly higher mean age, follicle-stimulating hormone, luteinizing hormone, UF number, UF volume, uterine volume, disease duration, gravidity, parity, and prolactin (PRL) levels compared with the myomectomy group (all *P* < .001). In contrast, estradiol and anti-Müllerian hormone levels were significantly lower in the hysterectomy group (*P* < .001). Across all modeling experiments, 2012 of 2555 model–input combinations achieved perfect classification performance (accuracy = 100%) when sex hormone profiles and UF characteristics were jointly used as inputs. Models using UF number alone also demonstrated high predictive performance, with accuracy reaching up to 96%. Agreement between algorithmic predictions and final surgical decisions was observed in 97% of cases, with one discordant case identified at a clinically borderline threshold. ML models trained on hormone profiles and fibroid characteristics were able to reproduce prevailing surgical classification patterns, largely reflecting strong baseline separability driven by age- and menopause-associated hormonal profiles, with consistent performance observed in an independent blinded validation cohort. These findings support the feasibility of quantitatively modeling routine decision structures, while highlighting the need for further validation in clinically heterogeneous and ambiguous cases.

## 1. Introduction

Uterine fibroids (UFs) are the most common benign myometrial tumors in women, with an estimated prevalence exceeding 50%.^[1,2]^ Most patients with UFs present with symptoms such as abnormal uterine bleeding, pelvic pain, obstetric complications, or infertility.^[3]^ Treatment options range from medical management to surgical interventions, depending on fertility desires, severity of symptoms, patient age, and comorbidities.^[4,5]^ For women not seeking fertility, hysterectomy remains the definitive treatment, whereas myomectomy is preferred in women wishing to preserve fertility.^[6]^

Surgical treatment options such as myomectomy and hysterectomy remain the conventional approaches for managing UFs. An increased mortality rate has been reported in patients younger than 50 years who underwent hysterectomy without prior estrogen therapy. In 1995, uterine artery embolization was first introduced into clinical practice as a less invasive alternative to surgical treatment.^[7]^ Subsequent evidence and guideline-based recommendations indicate that this procedure is now frequently performed as an elective option for UFs.^[8]^ When uterine preservation is desired, myomectomy is considered the standard of care, although the most appropriate approach remains uncertain. Although several guidelines have been published to aid decision-making for UF management, risks should always be discussed with the patient before intervention.^[9–12]^ Furthermore, factors such as surgeon expertise and institutional experience with current techniques must also be considered.

Consequently, several surgical interventions are available for UFs, including hysterectomy, hysteroscopic myomectomy, and myomectomy performed via laparotomy or laparoscopy.^[13]^ The patient fertility expectations play a critical role in determining the management strategy. Hysterectomy is generally avoided in women who wish to preserve fertility unless absolutely necessary. Female sex hormone levels, routinely considered during fertility assessment, are closely linked to surgical approach selection through their strong association with age and menopausal status. For example, perimenopausal or postmenopausal women typically present with low anti-Müllerian hormone (AMH) levels and elevated follicle-stimulating hormone (FSH) and luteinizing hormone (LH) levels.^[14,15]^ These hormonal profiles therefore function not only as biological markers of reproductive potential, but also as clinical proxies for menopausal transition, which itself plays a central role in surgical decision-making. Consequently, any predictive modeling approach relying solely on individual hormonal parameters may primarily capture hormonally mediated, age-related decision patterns rather than independent causal effects of specific hormones. For this reason, understanding surgical classification requires a comprehensive, multivariable analytical framework in which hormonal parameters are evaluated together with fibroid-related characteristics, including fibroid number, size, uterine volume, and disease burden. A multi-input analytical strategy is therefore essential to appropriately interrogate the complex and interdependent structure of real-world surgical classification in UF management.

However, there is a lack of quantitative, data-driven models that can integrate patient-specific hormone profiles and fibroid characteristics to objectively predict the most likely surgical outcome, which could standardize decision-making and support clinicians. The use of machine learning (ML) in this context is motivated by its capacity to integrate and analyze complex, nonlinear interactions among multiple clinical variables that jointly influence surgical classification. Unlike traditional statistical approaches, which often rely on linear assumptions or isolated variable effects, ML techniques can model higher-order relationships, for example, how age-related hormonal changes, AMH levels, and fibroid burden interact simultaneously to shape routine surgical classifications. In addition, ML methods enable the systematic identification of latent patterns embedded in multidimensional clinical data that may not be readily apparent through conventional analytical frameworks. When applied in a carefully constrained and interpretable manner, such models can reproduce individualized classification tendencies observed in real-world practice, thereby moving beyond population-level summaries to a structured representation of patient-specific decision contexts without replacing clinical judgment.

Furthermore, artificial intelligence (AI) models may provide valuable support for clinicians with limited experience in this field by facilitating more accurate and efficient decision-making. The present study therefore aimed to explore whether AI-based models could formally characterize surgical classification patterns, hysterectomy versus myomectomy, using fibroid characteristics and female sex hormone profiles.

## 2. Materials and methods

### 2.1. Ethical consideration

This study was conducted in accordance with the principles of the declaration of Helsinki and was approved by the Institutional Review Board of the Beykoz State Hospital Ethics Committee (protocol code: BEYKOZ DH 52; date of approval: August 09, 2022).

### 2.2. Patients

Initially, 645 patients with UFs were evaluated, but 45 were excluded due to insufficient data. Consequently, a total of 600 patients who presented to the gynecology and obstetrics departments of Private Derindere Hospital, Ataköy Medicana Hospital, and Kizilay Kağithane Hospital in Turkey between 2019 and 2024 were included in this study. Among them, 362 patients (60.3%) underwent hysterectomy, while 238 patients (39.7%) underwent myomectomy. Statistical analyses were planned and conducted separately for the hysterectomy and myomectomy groups.

### 2.3. Study design

This was a national, multicenter, retrospective study. Clinical and laboratory findings potentially associated with UFs, as presented in the descriptive table (Table 1), were included in the analyses. Patients with incomplete medical records or missing hormonal/laboratory data were excluded from the study, and only complete datasets were used for statistical and ML analyses. Statistical analyses and ML modeling were performed separately for the hysterectomy and myomectomy groups. ML training was conducted using female sex hormones, estradiol, FSH, LH, prolactin (PRL), and AMH, in combination with UF characteristics, through multiple input analyses.

**Table 1:** Case characteristics and comparative results between hysterectomy and myomectomy groups.

	Hysterectomy mean	Myomectomy mean	*P*-value
Patient *N*, %	238, (39.7)	362, (60.3)	
Age	48.6	35.7	<.001
FSH (mIU/mL)	56.2	7.3	<.001
LH (mIU/mL)	40.5	6.1	<.001
E2 (mIU/mL)	14.8	47	<.001
PRL (µg/L)	17.6	10.6	<.001
AMH (ng/mL)	0.2	15.7	<.001
Fibroid number	8	4.7	<.001
Fibroid volume (cc)	145	91	<.001
Uterus volume (cc)	243	92	<.001
Disease duration			<.001
1–5 years	0 (0%)	126 (53%)	
>5 years	362 (100%)	112 (47%)	
Gravidity			<.001
Yes	362 (100%)	212 (89%)	
No	0 (0%)	26 (11%)	
Parity			<.001
Yes	362 (100%)	148 (62%)	
No	0 (0%)	90 (38%)	

AMH = antimüllerian hormone, E2 = estradiol, FSH = follicle stimulating hormone, LH = luteinizing hormone, PRL = prolactin.

The performance of the selected best-performing model was further evaluated using an independent, blinded external validation cohort comprising 30 cases. Agreement between algorithmic predictions and final surgical decisions was subsequently assessed.

### 2.4. Data preprocessing

Prior to statistical analysis and ML modeling, a standardized data preprocessing pipeline was applied to ensure consistency and analytical integrity. Only complete cases were included in the final dataset; patients with missing clinical, hormonal, or fibroid-related variables were excluded from the analyses. This approach was chosen to avoid bias introduced by data imputation and to maintain clinical interpretability of all input features.

All continuous variables were examined for distributional characteristics using skewness, kurtosis, and normality testing. No artificial transformation or discretization of hormone or fibroid-related parameters was performed, as the aim was to preserve their original clinical scale and real-world interpretability. Categorical variables were handled in their original binary or nominal form as appropriate.

For ML analyses, the same preprocessing steps were applied uniformly to the training, testing, and external validation datasets to prevent data leakage and ensure comparability across model evaluations. Data splitting was performed after preprocessing, and no information from the test or validation sets was used during model training.

### 2.5. Class distribution and handling of imbalance

The distribution of surgical classes was moderately imbalanced, with 362 hysterectomy cases and 238 myomectomy cases. No synthetic oversampling or class re-weighting techniques (e.g., SMOTE or cost-sensitive learning) were applied. This decision was intentional, as the primary objective of the study was not to optimize predictive performance under artificially balanced conditions, but rather to model and reproduce real-world clinical classification patterns observed in routine practice.

To mitigate potential bias, training and test splits were generated using stratified sampling to preserve the original class proportions across datasets. Moreover, model performance was evaluated using multiple complementary metrics beyond accuracy, including sensitivity, precision, F1 score, and area under the receiver operating characteristic curve. Finally, external blinded validation served as an additional safeguard against majority-class bias by testing model behavior on independent cases.

### 2.6. Feature scaling

Feature scaling (normalization or standardization) was not applied prior to model training. This decision was made intentionally to preserve the original clinical scale and interpretability of hormonal and fibroid-related variables, which are routinely evaluated in absolute units in daily practice. In particular, the study aimed to model real-world surgical classification patterns rather than to optimize abstract algorithmic performance.

Algorithms inherently robust to feature scale, such as random forest and decision tree models, constituted a substantial proportion of the modeling framework. For scale-sensitive algorithms (e.g., support vector machines and k-nearest neighbors), model stability was assessed through consistent performance across training, test, cross-validation, and blinded external validation analyses, suggesting that lack of feature scaling did not materially affect the primary conclusions of the study.

### 2.7. Hyperparameter configuration

ML models were trained using default hyperparameters without additional tuning. This approach was chosen to prioritize clinical interpretability and to assess whether observed performance reflected intrinsic data structure rather than optimization-driven effects. Model stability was evaluated using cross-validation and external validation procedures.

### 2.8. Validation procedure

To evaluate the external generalizability of the developed ML models, an independent validation analysis was performed using 30 anonymized clinical cases. These cases were analyzed following the same preprocessing pipeline used in the primary analyses. Predictions were generated without access to surgical outcomes. In parallel, an experienced gynecologist independently reviewed the same cases based solely on the available clinical and hormonal parameters and classified each case according to the surgical approach that had ultimately been performed. Both the clinician and the model analyses were conducted in a blinded manner. Agreement between algorithmic predictions and clinical classifications was subsequently assessed.

### 2.9. Statistical analyses and tools

Statistical analyses and ML training were performed using Wistats v3.0 (WisdomEra Corp., Istanbul, Turkey), which incorporates Python-based statistical and ML libraries (SciPy v1.2.3, scikit-learn v0.24.0, statsmodels v0.9.0). Data distribution was evaluated using skewness and kurtosis, and normality was assessed with the Shapiro–Wilk test. The choice of comparative statistical tests was determined according to data distribution. Chi-square and Fisher exact tests were applied for categoricalvariables. Kruskal–Wallis, one-way ANOVA, independent-samples *t*-test, and Mann–Whitney *U* tests were used for comparisons involving categorical and numerical data. Pearson and Spearman correlation analyses were performed to assess associations between continuous variables. A *P*-value < .05 was considered statistically significant.

### 2.10. ML procedure and pipeline

**Figure 1.**
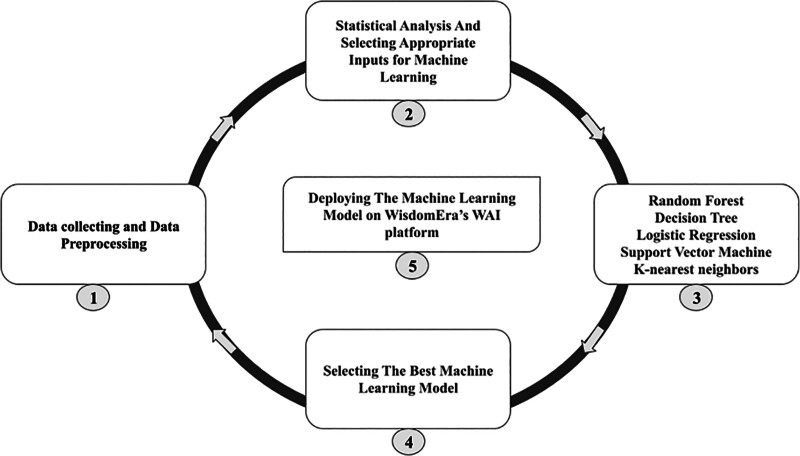
Machine learning procedure and pipeline. This figure illustrates the complete workflow used to develop the clinical decision support framework. The process begins with data collection and preprocessing, followed by statistical analyses to identify meaningful hormonal and fibroid-related predictors. Five supervised learning algorithms, random forest, decision tree, logistic regression, support vector machine, and k-nearest neighbors, were trained using multiple input combinations. The model with the highest predictive performance was selected and subsequently deployed on the WisdomEra artificial intelligence platform for real-time clinical decision support. This figure demonstrates the stepwise transformation of raw clinical data into a functional and deployable computational tool.

Statistical analyses were conducted prior to the development of ML models. The surgical type (hysterectomy or myomectomy) was defined as the output variable. Five classification algorithms were applied: random forest (RF), support vector machine, k-nearest neighbors (KNN), decision tree, and logistic regression (LR). A 70:30 ratio was used for training and test data, with 30% allocated as the test set. Model performance was evaluated using multiple metrics, including the area under the curve, accuracy, sensitivity, precision, and F1 score (Fig. 1).

### 2.11. Overfitting assessment

To explore whether the high predictive performance reflected model overfitting, multiple complementary analyses were performed. First, training and testing accuracies were compared for each model configuration, and the absence of large discrepancies was considered supportive, but not definitive, evidence against substantial overfitting.

Second, 5-fold cross-validation was conducted within the training data to obtain a more stable estimate of performance across data partitions. Consistent accuracy and area under the receiver operating characteristic curve values across folds and between cross-validation and hold-out testing suggested that model performance was not solely driven by a single data split.

Nevertheless, given the pronounced clinical separation between hysterectomy and myomectomy cohorts, high classification accuracy may partially reflect underlying data separability rather than purely model complexity. Accordingly, these analyses support model stability while acknowledging that predictive performance may be influenced by cohort structure.

### 2.12. Model visualization and research interface

**Figure 2.**
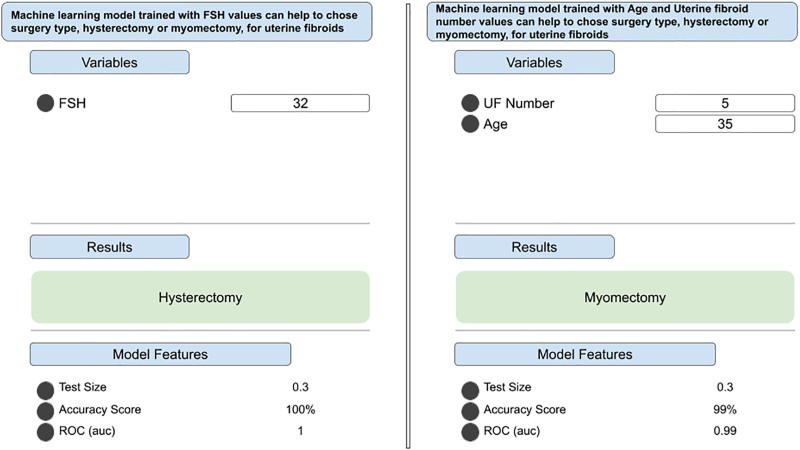
Research-oriented visualization interface for machine learning (ML)–based surgical classification. This figure illustrates 2 representative examples of the graphical user interface developed for research visualization of ML model outputs. The left panel demonstrates a model trained using a single hormonal input (follicle-stimulating hormone), while the right panel shows a model using combined inputs of patient age and uterine fibroid number. For each example, user-defined input variables are entered under the predefined modeling configuration, and the resulting surgical classification (hysterectomy or myomectomy) is displayed. Model-related summary information, including test dataset proportion, accuracy score, and area under the receiver operating characteristic curve, is presented to facilitate interpretability of model behavior. This interface was used exclusively for exploratory analysis and demonstration purposes within the study and does not represent a clinically deployed or prospectively implemented decision-support system. ML = machine learning.

For research visualization purposes, the model with the highest performance was integrated into a web-based analytical interface, enabling exploratory inspection of input–output behavior. This interface was used solely for demonstration and analysis within the scope of this study and did not constitute clinical implementation or prospective decision support (Fig. 2).

## 3. Results

### 3.1. Statistical results

The detailed descriptive statistics and comparative statistical results are presented in Table 1. The mean age value of the hysterectomy group was found to be statistically significantly higher than the myomectomy group (48.6, 35.7, respectively; *P* < .001). The percentage of disease duration (>5 years) of the hysterectomy group was found to be statistically significantly higher than the myomectomy group (100%, 47%, respectively; *P* < .001). The percentage of gravidity of the hysterectomy group was found to be statistically significantly higher than the myomectomy group (100%, 89%, respectively; *P* < .001). The percentage of parity of the hysterectomy group was found to be statistically significantly higher than the myomectomy group (100%, 62%, respectively; *P* < .001).

The FSH, LH, and PRL values of the hysterectomy group were found to be statistically significantly higher than the myomectomy group (*P*-value in all comparisons: <.001). The estradiol and AMH values of the hysterectomy group were found to be statistically significantly lower than the myomectomy group (*P*-value in all comparisons: <.001).

The mean of UF number and UF volume values of the hysterectomy group were found to be statistically significantly higher than the myomectomy group (*P*-value in all comparisons: <.001).

### 3.2. ML model training results

**Figure 3.**
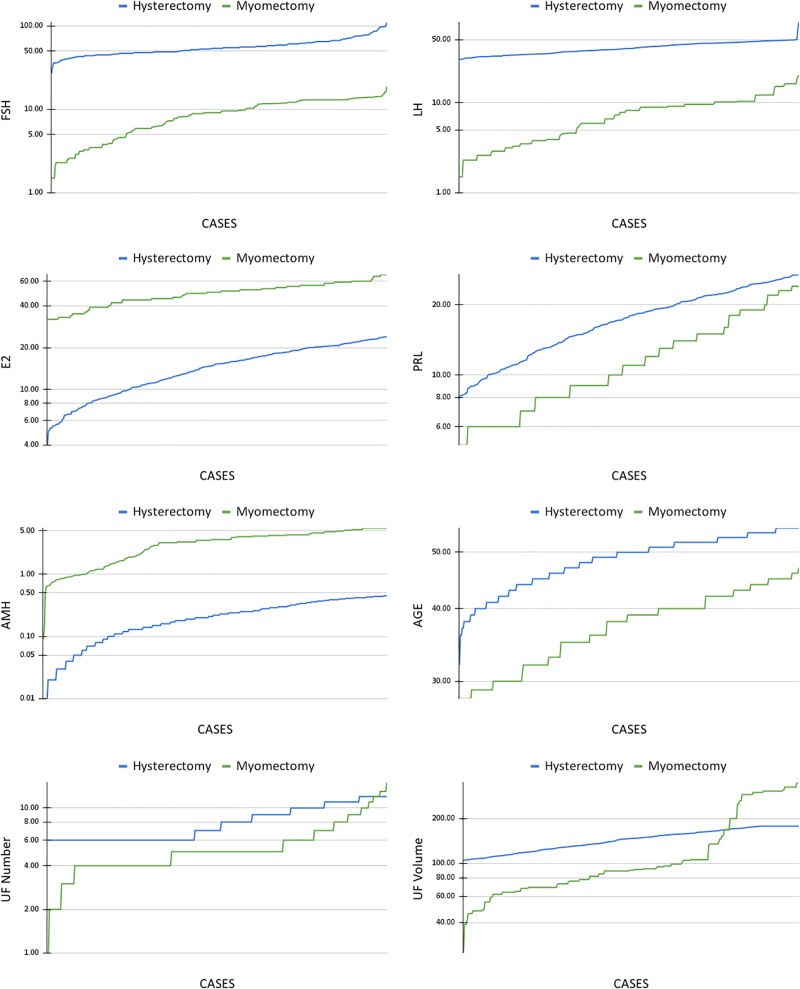
Case-based visualization of hormone profiles, fibroid characteristics, and age distributions across surgical groups. This figure presents sorted case-based distributions (from minimum to maximum values) of key clinical, hormonal, and fibroid-related variables stratified by surgical treatment group (hysterectomy vs. myomectomy). Panels illustrate follicle-stimulating hormone, luteinizing hormone, estradiol, prolactin, anti-Müllerian hormone, patient age, uterine fibroid number, and UF volume. Across hormonal parameters and age, the two surgical cohorts demonstrate minimal overlap, with distribution curves rarely intersecting, indicating pronounced baseline clinical separation largely driven by age- and menopause-associated hormonal profiles. Fibroid-related variables, particularly UF number and UF volume, also show higher values in the hysterectomy group, further contributing to cohort distinction. This visualization provides a structural explanation for the high classification accuracy observed in machine learning analyses, highlighting that model performance primarily reflects reproducible differences in underlying data distributions rather than complex algorithmic inference. ML = machine learning, UF number = uterine fibroid number, UF volume = uterine fibroid volume.

A total of 2555 ML trainings with different input and ML model combinations were performed. We identified 2012 distinct ML models that achieved a 100% accuracy rate when female sex hormone parameters and UF characteristics were jointly used as inputs. To investigate the underlying reasons for these very high accuracy rates, we examined the data distribution using line charts comparing hormone values between the hysterectomy and myomectomy groups (Fig. 3). Across all female sex hormone parameters, the distributions of the 2 surgical cohorts demonstrated minimal overlap, with the corresponding curves rarely intersecting. This pronounced separation at the feature level provides a structural explanation for the observed classification performance and indicates that model accuracy was driven primarily by clear baseline differences between cohorts rather than by complex algorithmic inference.

Importantly, the close agreement observed between training, test, and cross-validation accuracy across all evaluated models further supports the stability of these findings and argues against model overfitting. Instead, the consistency across multiple resampling conditions suggests that the learned decision boundaries reflect reproducible patterns present in the underlying data structure.

In addition to hormonal parameters, ML models developed using UF number as a single input variable also demonstrated high predictive performance, with accuracy reaching up to 96%. Representative examples of these models are presented in Table 2. All trained ML models were subsequently categorized according to accuracy thresholds (100%, >90%, and <90%), and the distribution of models across these performance groups is summarized in Table 3.

**Table 2:** Examples of machine learning models with different input combinations by female sex hormone values and UF characteristics.

Inputs	Model	Accuracy	ROC	Precision	Recall	*F* score
FSH	SVM	1	1	1	1	1
LH	KNN	1	1	1	1	1
E2	LR	1	1	1	1	1
AMH	RF	1	1	1	1	1
FSH, age, UF number	DT	1	1	1	1	1
LH, age, UF number	SVM	1	1	1	1	1
Age, UF number	DT	0.99	0.99	0.99	0.99	0.99
Age, UF volume	KNN	0.98	0.97	0.99	0.96	0.97
Age	LR	0.90	0.95	0.93	0.80	0.86
UF number	RF	0.96	0.94	1	0.88	0.94
UF volume	LR	0.96	0.94	1	0.88	0.94

Performance metrics were calculated on the independent test set (30% of the total cohort).

AMH = anti-Müllerian hormone, DT = decision tree, E2 = estradiol, FSH = follicle-stimulating hormone, KNN = k-nearest neighbors, LH = luteinizing hormone, LR = logistic regression, RF = random forest, SVM = support vector machine, UF number = uterine fibroid number, UF volume = uterine fibroid volume.

**Table 3:** Number of machine learning models based on accuracy rate groups.

Accuracy ratio group, %	Count, %
100	2012 (78.7%)
90–100	538 (21%)
<90	5 (0.3%)
Total	2555

This table summarizes the distribution of all trained machine learning models grouped by accuracy thresholds (100%, >90%, and < 90%) across different algorithm and input-variable combinations. The high proportion of models achieving perfect accuracy reflects pronounced baseline separability between hysterectomy and myomectomy cohorts, as demonstrated in Fig. 3, rather than superior algorithmic complexity. Accordingly, the table illustrates the robustness and reproducibility of cohort-level classification patterns across multiple modeling configurations, rather than the resolution of clinically ambiguous surgical decision scenarios.

### 3.3. External validation results

Concordant classifications were observed in 29 of the 30 cases, corresponding to an agreement rate of 97%. The single discordant case was subsequently reviewed and identified as a clinically borderline scenario, in which surgical decision-making is inherently complex.

This level of agreement indicates that the model was able to reproduce prevailing clinical classification patterns in an independent dataset, supporting their generalizability while underscoring the need for further evaluation in cohorts enriched with clinically ambiguous cases.

## 4. Discussion

Surgical treatments such as hysterectomy and myomectomy remain the conventional approaches for managing UFs.^[16]^ UFs substantially affect quality of life, primarily through abnormal uterine bleeding and the consequent development of iron deficiency anemia.^[17]^ The patient’s fertility expectations play a critical role in determining the management strategy. Hysterectomy is generally avoided in women who wish to preserve fertility and is performed only when clinically indicated. Female sex hormone levels, routinely considered during fertility assessment, are closely associated with the type of surgical intervention. In this study, ML models achieved high predictive accuracy when trained on combinations of hormonal parameters and UF characteristics. Notably, perfect classification was observed in a substantial proportion of model–input combinations, and UF number alone also demonstrated strong predictive performance. Rather than reflecting superior algorithmic complexity, these findings primarily reveal a pronounced clinical separation between hysterectomy and myomectomy cohorts, driven by age-related and hormonally mediated biological differences. Accordingly, the high accuracy achieved by several models should be interpreted as the ability to formalize and reproduce prevailing clinical classification patterns, rather than as evidence of resolving complex or ambiguous surgical dilemmas. The additional external blinded validation analysis further supported this interpretation, showing high concordance while identifying discordant cases predominantly within clinically borderline scenarios. Together, these findings suggest that the models capture established decision contours in fibroid management, highlighting both their methodological consistency and their current limitations.

Guidelines for the medical and surgical management of UFs have been well established in the literature.^[10,11]^ Several medical treatment options have been shown to effectively reduce UF size and alleviate UF-related symptoms. Currently, gonadotropin-releasing hormone (GnRH) agonists, which induce hypoestrogenism, are routinely used as a medical therapy to shrink UFs. In symptomatic cases, hysterectomy remains the definitive treatment approach. Myomectomy, on the other hand, may be preferred in women who wish to preserve the uterus or maintain fertility potential. In our study, the parity rate was significantly higher in the hysterectomy group compared with the myomectomy group (100% vs. 62%, *P* < .001). Myomectomy should therefore be reserved for symptomatic cases. Importantly, physicians must inform patients about the potential consequences of myomectomy on future fertility.

In the perimenopausal period, hysterectomy is considered the most effective treatment for symptomatic UFs and is associated with high patient satisfaction rates.^[10,11]^ In our study, hysterectomy was predominantly performed in perimenopausal or postmenopausal women (minimum age: 32 years; 78% of hysterectomy cases were > 45 years). The mean age of patients undergoing hysterectomy was significantly higher compared with those undergoing myomectomy (48.6 vs. 35.7 years, *P* < .001). In addition, both UF volume and UF number were significantly greater in the hysterectomy group than in the myomectomy group, reflecting the higher symptom burden.

ML and AI, as leading technologies of today, offer promising opportunities for the future by helping to overcome diagnostic challenges, personalize treatment strategies, and improve patient outcomes.^[18]^ The potential role of AI as an assistant to support clinical experts has been increasingly explored across various medical fields. In the literature, several image-processing–based AI models have been developed to aid in the diagnosis and treatment of UFs. For example, Huo and colleagues evaluated an AI model designed to assist junior ultrasonographers in improving the diagnostic accuracy of UFs and compared its performance with that of senior ultrasonographers, thereby confirming the effectiveness and feasibility of the AI approach. The AI model enabled junior clinicians to achieve higher diagnostic accuracy (94.72% vs. 86.63%).^[19]^ In our study, we transferred clinical practice experiences into AI models by training them with real-world data using ML algorithms. This approach may enable junior gynecologists to provide more effective treatment decisions regarding the necessity and timing of surgery.

The present study aimed to explore whether routinely available hormonal and fibroid-related parameters could be used to formally model surgical classification patterns observed in real-world clinical practice. Rather than replacing clinical judgment, such models may offer complementary value for educational purposes, research standardization, and the study of decision variability, particularly among clinically clear-cut versus borderline cases.

This study has several limitations that warrant consideration. First, the dataset did not include patients receiving medical (non-surgical) management, restricting the analysis to surgical cohorts and limiting the ability to model decision-making across the full spectrum of UF treatment pathways. Incorporating medical therapy groups in future studies may enable the development of more comprehensive models capturing broader clinical trajectories. Second, the retrospective design renders the analyses susceptible to unmeasured confounding, particularly given the strong association between age, hormonal profiles, and surgical choice. The pronounced clinical separation between hysterectomy and myomectomy cohorts likely contributed to the high classification accuracy observed and limits the applicability of these models in clinically ambiguous scenarios. Although external blinded validation supported model generalizability, these algorithms should currently be interpreted as research tools that formalize existing clinical classification patterns.

Furthermore, our literature review revealed no prior ML-based decision-support algorithms developed specifically with female sex hormone parameters and fibroid characteristics to predict surgical type. To the best of our knowledge, this is the first study addressing this gap in the literature.

## 5. Conclusions

In conclusion, female sex hormone profiles were strongly associated with the surgical management patterns observed in women with UFs, reflecting their close relationship with age, reproductive status, and disease characteristics. Using these routinely available parameters, ML models were able to reproduce prevailing clinical classification patterns distinguishing hysterectomy from myomectomy. Model performance remained consistent in an independent, blinded external validation cohort, supporting the generalizability of the learned patterns while also highlighting that predictive accuracy was influenced by the underlying clinical separation between patient groups. Accordingly, these findings should be interpreted as a proof-of-concept demonstrating the feasibility of quantitatively modeling routine surgical decision structures rather than as evidence of causal inference or immediate clinical applicability. Future studies should focus on external validation in more heterogeneous cohorts, particularly those enriched with clinically ambiguous cases, to better define the contexts in which such models may provide complementary support for education, decision standardization, or research purposes.

## Acknowledgments

We would like to thank the Artificial Intelligence Research and Development Center of Istinye University (https://yzaum.istinye.edu.tr/) for the article assessment in terms of article structure and data analytics details. We would like to thank the Ditako Corporation Team (https://ditako.com) for data analytics and software development services.

## Author contributions

**Conceptualization:** İnci Öz, Ali Utku Öz.

**Data curation:** İnci Öz, Ali Utku Öz.

**Investigation:** İnci Öz, Ali Utku Öz.

**Methodology:** İnci Öz, Ali Utku Öz.

**Resources:** İnci Öz, Ali Utku Öz.

**Supervision:** İnci Öz.

**Writing – original draft:** İnci Öz.

**Writing – review & editing:** İnci Öz, Ali Utku Öz.

## References

[1] Meyer R Hamilton KM Schneyer RJ. Short-term outcomes of minimally invasive total vs supracervical hysterectomy for uterine fibroids: a national surgical quality improvement program study. Am J Obstet Gynecol. 2025;232:377.e1–377.e10. 10.1016/j.ajog.2024.10.006 39413898

[2] Day Baird D Dunson DB Hill MC Cousins D Schectman JM. High cumulative incidence of uterine leiomyoma in black and white women: ultrasound evidence. Am J Obstet Gynecol. 2003;188:100–7. 12548202 10.1067/mob.2003.99

[3] Donnez J Dolmans M-M. Uterine fibroid management: from the present to the future. Hum Reprod Update. 2016;22:665–86. 27466209 10.1093/humupd/dmw023 PMC5853598

[4] Donnez J Taylor HS Marcellin L Dolmans M-M. Uterine fibroid–related infertility: mechanisms and management. Fertil Steril. 2024;122:31–9. 38453041 10.1016/j.fertnstert.2024.02.049

[5] Anchan RM Spies JB Zhang S. Long-term health-related quality of life and symptom severity following hysterectomy, myomectomy, or uterine artery embolization for the treatment of symptomatic uterine fibroids. Am J Obstet Gynecol. 2023;229:275.e1–275.e17. 10.1016/j.ajog.2023.05.020 37244458

[6] Cianci S Gulino FA Palmara V. Exploring surgical strategies for uterine fibroid treatment: a comprehensive review of literature on open and minimally invasive approaches. Medicina (Mex). 2024;60:64. 10.3390/medicina60010064 PMC10820219 38256325

[7] Ravina JH Herbreteau D Ciraru-Vigneron N. Arterial embolisation to treat uterine myomata. Lancet (London, England). 1995;346:671–2. 7544859 10.1016/s0140-6736(95)92282-2

[8] Amoah A Joseph N Reap S Quinn SD. Appraisal of national and international uterine fibroid management guidelines: a systematic review. BJOG. 2022;129:356–64. 34532956 10.1111/1471-0528.16928

[9] American College of Obstetricians and Gynecologists. ACOG practice bulletin. Alternatives to hysterectomy in the management of leiomyomas. Obstet Gynecol. 2008;112:387–400. 18669742 10.1097/AOG.0b013e318183fbab

[10] Practice Committee of American Society for Reproductive Medicine in collaboration with Society of Reproductive Surgeons. Myomas and reproductive function. Fertil Steril. 2008;90:S125–130. 19007608 10.1016/j.fertnstert.2008.09.012

[11] Marret H Fritel X Ouldamer L; CNGOF (French College of Gynecology and Obstetrics). Therapeutic management of uterine fibroid tumors: updated French guidelines. Eur J Obstet Gynecol Reprod Biol. 2012;165:156–64. 22939241 10.1016/j.ejogrb.2012.07.030

[12] Stewart EA. Clinical practice. Uterine fibroids. N Engl J Med. 2015;372:1646–55. 25901428 10.1056/NEJMcp1411029

[13] Donnez J Jadoul P. What are the implications of myomas on fertility? A need for a debate? Hum Reprod. 2002;17:1424–30. 12042254 10.1093/humrep/17.6.1424

[14] Kawakita T Yasui T Yoshida K Matsui S Iwasa T. Associations of LH and FSH with reproductive hormones depending on each stage of the menopausal transition. BMC Womens Health. 2023;23:286. 37231423 10.1186/s12905-023-02438-5 PMC10214553

[15] Freeman EW Sammel MD Lin H Gracia CR. Anti-Mullerian hormone as a predictor of time to menopause in late reproductive age women. J Clin Endocrinol Metab. 2012;97:1673–80. 22378815 10.1210/jc.2011-3032 PMC3339896

[16] Parker WH Feskanich D Broder MS. Long-term mortality associated with oophorectomy compared with ovarian conservation in the nurses’ health study. Obstet Gynecol. 2013;121:709–16. 23635669 10.1097/AOG.0b013e3182864350 PMC4254662

[17] Vannuccini S Petraglia F Carmona F Calaf J Chapron C. The modern management of uterine fibroids-related abnormal uterine bleeding. Fertil Steril. 2024;122:20–30. 38723935 10.1016/j.fertnstert.2024.04.041

[18] Polat G Arslan HK. Artificial intelligence in clinical and surgical gynecology. İstanbul Gelişim Üniv Sağlik Bilim Derg. 2024;21:1232–41.

[19] Huo T Li L Chen X. Artificial intelligence-aided method to detect uterine fibroids in ultrasound images: a retrospective study. Sci Rep. 2023;13:3714. 36878941 10.1038/s41598-022-26771-1 PMC9988965

